# The association between systemic immune-inflammation index and in vitro fertilization outcomes in women with polycystic ovary syndrome: a cohort study

**DOI:** 10.1186/s13048-023-01321-z

**Published:** 2023-12-13

**Authors:** Xin Li, Ting Luan, Yi Wei, Juan Zhang, JuanJuan Zhang, Chun Zhao, Xiufeng Ling

**Affiliations:** 1 Department of Reproductive Medicine, Women’s Hospital of Nanjing Medical University, Nanjing Maternity and Child Health Care Hospital, Nanjing, China; 2 Department of Obstetrics and Gynecology, Women’s Hospital of Nanjing Medical University, Nanjing Maternity and Child Health Care Hospital, Nanjing, China

**Keywords:** Assisted reproductive technology, Polycystic ovary syndrome, Systemic immune-inflammation index, in vitro fertilization outcomes

## Abstract

**Background:**

As a novel prognostic and inflammatory marker, the systemic immune-inflammation index (SII) has come to the foreground in recent years. SII may be used as an indicator reflecting the progressive inflammatory process in patients with polycystic ovary syndrome (PCOS). This study aimed to evaluate the correlation between SII and assisted reproductive outcomes in PCOS patients.

**Results:**

A total of 966 women undergoing in vitro fertilization (IVF) procedure with PCOS were included in the study. The SII was calculated as platelet count (/L) × neutrophil count (/L)/lymphocyte count (/L). Participants were divided into four groups according to SII quartiles calculated at baseline, and the differences of clinical and laboratory outcomes between these four groups were compared. Moreover, a univariate linear regression model was used to evaluate the associations between SII and the outcomes. Patients in the highest SII quartile (Q4) had lower antral follicle count (AFC), estradiol (E2), and progesterone (P) levels on the day of human chorionic gonadotropin (HCG) start compared with the lower three SII quartiles (Q1-Q3). Moreover, our analysis demonstrated that women in the lower SII quartiles had a higher rate of available embryos and blastocyst formation compared with those in the highest SII quartile. Logarithm of SII correlated negatively with available embryo rate, but not with number of available embryos. Additionally, the results of our multivariate logistic regression analyses indicated that the highest SII quartile was negatively associated with biochemical pregnancy rate (BPR), clinical pregnancy rate (CPR), live birth rate (LBR), and implantation rate (IR). A non-linear relationship between the SII and number of available embryos, with a negative relationship seen to the right of the inflection point was also found.

**Conclusions:**

The interplay among thrombocytosis, inflammation, and immunity could influence assisted reproductive outcomes in PCOS patients. In this regard, SII may serve as a valuable marker for exploring potential correlations.

**Supplementary Information:**

The online version contains supplementary material available at 10.1186/s13048-023-01321-z.

## Introduction

Polycystic ovary syndrome (PCOS) is a primary contributor to female infertility, playing a pivotal role in reproductive challenges for women. It affects a substantial proportion of women of reproductive age worldwide, with prevalence rates ranging from 6 to 20% [1, 2]. PCOS is generally characterized by anovulation or oligo-anovulation, androgen excess and ovarian polycystic morphology [3]. The diagnosis of PCOS, as per the Rotterdam criteria, requires the presence of a minimum of two out of the following three features: oligoanovulation, clinical and/or biochemical hyperandrogenism, and polycystic ovaries observed through ultrasonography, while excluding other endocrinopathies. The clinical manifestations of PCOS are complicated and individualized and the precise etiology remains elusive. Current research suggests that the etiology of PCOS may be multifactorial, possibly related to reactive oxygen species (ROS) [4], inflammatory reactions [5, 6], genetic predisposition [7], excessive exposure to embryonic androgens [8], unhealthy lifestyle factors [9], and hormonal disorders[10]. In addition, there is growing recognition that low-grade chronic inflammation plays a crucial role as both a symptom and a contributing factor [11, 12]. In recent years, an increasing number of studies have focused on the critical role of inflammation in PCOS. Previous studies reported that significantly higher concentrations of inflammatory cells were detected in the peripheral blood of PCOS patients. Leukocytosis, neutrophilia, and platelet aggregation have been frequently detected in PCOS patient blood profile [13, 14]. The systemic immune-inflammation index (SII) is a novel index based on the lymphocyte, neutrophil, and platelet counts. A mounting body of evidence illustrates that SII is a useful index to reflect the systemic immune and inflammatory status of the human body [15–17]. SII has been reported to be associated with various inflammatory and reproductive disorders, such as endometrial cancer, ovarian cancer [18, 19]. To the best of our knowledge, no study has explored the association between SII and in vitro fertilization (IVF) outcomes in women with PCOS. First introduced by Hu et al. [20], this marker has been demonstrated to gauge the severity of systemic inflammation in carcinoma patients [21] and offers high prognostic value across various cancer types [22], where local and systemic inflammation are key features [23].

Women with PCOS often seek the help of assisted reproductive technology (ART) to get pregnant due to disorders of oocyte maturation or any other male or female factors. In recent years, gonadotropin releasing hormone antagonist (GnRH-ant) protocol has been widely used among PCOS patients, due to its short stimulation duration, low gonadotropin consumption and significantly lower incidence of ovarian hyperstimulation syndrome (OHSS) [24]. As SII integrates the information of neutrophil, platelet and lymphocyte counts, the analysis of these three types of blood cells together could elucidate their interaction in the pathological process of PCOS, though their possible opposite roles in the process may not be appreciated. Furthermore, it would be of greater value to elaborate the potential interactive effects of platelets, lymphocytes and neutrophils on PCOS women. To address these issues, our study aimed to investigate the relationship between the SII on the day before oocyte retrieval and the outcomes of assisted conception in women diagnosed with PCOS who underwent treatment with GnRH-ant protocol.

## Materials and methods

### Study design and participants

This study was a hospital-based cohort study, carried out at Women’s Hospital of Nanjing Medical University between January 2018 and September 2020. PCOS patients who underwent their first IVF/ Intracytoplasmic sperm injection (ICSI)-embryos transfer (ET) were evaluated. Inclusion criteria: (1) Patients aged between 20 and 40 years, diagnosed with PCOS according to the Rotterdam diagnostic criteria (3), therefore fulfilling ≥ 2 of the following: oligo- or anovulation; clinical or biochemical signs of hyperandrogenism; and polycystic ovaries and exclusion of other etiologies (i.e., congenital adrenal hyperplasia, androgen-secreting tumors, or Cushing syndrome; (2) IVF or ICSI was used for insemination; (3) PCOS protocols were GnRH-ant protocol. Exclusion criteria: (1) Infertility patients caused by non-PCOS ovulatory disorder or other factors; (2) Patients with history of ovarian surgery or complication with endometriosis or pelvic adhesion; (3) Patients complicated with liver, kidney or thyroid dysfunction; (4) Recurrent spontaneous abortion (defined as three or more previous spontaneous pregnancy losses), congenital or acquired uterine malformations, abnormal parental karyotypes or medical conditions that contraindicated ART and/or pregnancy.

### Ovarian stimulation

Participants underwent a flexible GnRH-ant protocol, briefly, patients were injected daily with 150–225 IU recombinant FSH (rFSH, Gonal-F, Merck Serono, Italy) from day 2 or 3 of the menstrual cycle, with daily 0.125 mg-0.25 mg GnRH-ant (Cetrorelix, Merck Serono, Darmstadt, Germany) being initiated once the largest follicle was > 12–14 mm in size. We monitored follicular growth by ultrasound scan and sex hormone levels [follicle-stimulating hormone (FSH), luteinizing hormone (LH), estradiol(E2), and progesterone(P)] and adjusted the dose of gonadotropin (Gn). When at least two follicles are larger than 18 mm, a 10,000 IU human chorionic gonadotrophin (hCG, Lizhu, China) injection was given to achieve final oocyte maturation, and oocyte retrieval was scheduled 36 h later. For women at high risk for OHSS, low doses of hCG (5,000 IU) were used to trigger ovulation, a minority of patients opt for a double trigger. Serum sex hormone levels and ET were measured on the trigger day.

### Oocyte retrieval and embryo transfer

The oocytes and embryos were cultured according to our previously published article [25]. Based on sperm quality, conventional IVF or ICSI was performed. All the embryos were in vitro fertilization and cultured for 3 days (D3) or 5 days (D5). Ultrasound guidance was used for all ET and performed 3 or 5 days after oocyte retrieval, with a maximum of 2 embryos to be transferred. During the luteal phase, 20 mg progesterone injection was injected twice a day from the first day after oocyte retrieval.

### Freeze-all

Indications for the freeze-all policy included a high risk of developing OHSS (women who were younger than 35 years old, use hCG for ovarian stimulation or had a high response to Gn, the number of follicles > 14 mm was more than 20 or E2 level was more than 5000 pg/ml on trigger day), inadequate endometrial thickness (< 7 mm), high P level (> 2.0 ng/ml) and some other conditions (such as high blood pressure, fever, individual preference).

### Definition of SII

Peripheral venous blood samples were collected one day before oocyte retrieval in all patients. The counts of peripheral neutrophils, lymphocytes and platelets were measured and analyzed by an automatic blood analyzer (five-category hematological analyzer XT 2000i, SYSMEX, Japan). The definition of SII was shown as follow: SII = platelet × neutrophil/lymphocyte counts [26]. SII was designed as exposure variable in our analysis. The SII value was also transformed to a logarithmic scale (Log SII) to minimize skewness of the underlying distribution.

### Outcome assessment

#### Clinical outcomes

The delivery of a viable infant was considered as the live birth. A biochemical pregnancy was defined as a positive hCG level without a gestational sac. The presence of a gestational sac with or without fetal heart activity, ectopic pregnancy and heterotopic pregnancy was regarded as clinical pregnancy. The implantation rate (IR) was defined as the number of gestational sacs divided by the number of embryos transferred. The early pregnancy loss rate (EPLR) was defined as the proportion of patients with a spontaneous termination of pregnancy.

### Laboratory outcomes

The following oocyte and embryo parameters were analyzed: number of oocytes retrieved, number of two pronucleus (2PN), number of 2PN cleavage embryos, cleavage rate, number of cleavage embryos, number of available embryos, available embryo rate, number of superior-quality embryos and blastocyst formation rate.

### Statistical analysis

In this study, participants were divided into four groups according to SII quartiles calculated at baseline. Continuous variables were presented as mean ± standard deviation for normal distribution and median (quartile) for skewed distribution, while categorical variables were described using frequency or percentage. To determine the statistical differences between means and proportions of the groups, the normal distribution was analyzed using the One-Way ANOVA, while the skewed distribution was analyzed using the Kruskal Wallis H test and categorical variables were analyzed using the chi-square test. Moreover, we used a univariate linear regression model to evaluate the associations between SII and the outcomes. The results were presented for both unadjusted, minimally adjusted, and fully adjusted analyses. For investigating the non-linear relationship between SII on clinical and laboratory outcomes, we used Generalized Additive Models (GAM). If the non-linear correlation was observed, a two-piecewise linear regression model was performed to calculate the threshold effect of the SII on clinical and laboratory outcomes in terms of the smoothing plot. When the ratio between SII on clinical and laboratory outcomes appears obvious in smoothed curve, recursive method calculates automatically the inflection point, where the maximum model likelihood will be used [27]. *P* < 0.05 was used for statistical significance. All statistical analyses were performed using statistical software packages R (http://www.R-project.org, The R Foundation) and EmpowerStats software (http://www.mpowerstats.com, X&Y Solution, Inc., Boston, MA).

## Results

### Baseline characteristics of four groups

**Fig. 1:**
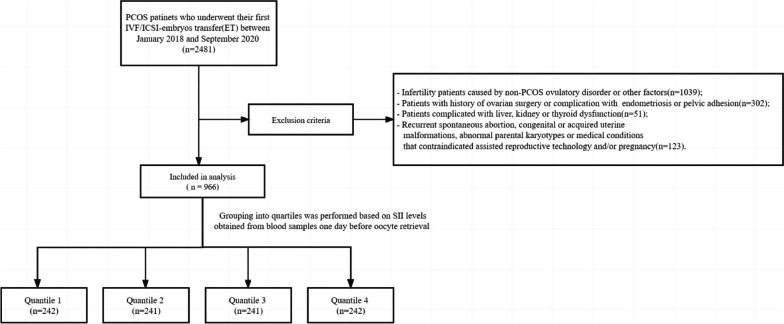
Flow-chart of the study cohort characteristics. PCOS polycystic Ovary Syndrome, SII Systematic immune-inflammation index

Figure 1 shows a flow chart of the study population. SII was divided into quartiles based on the distribution of baseline SII in the participants ((quartile 1 (Q1): < 776.63, quartile 2 (Q2): 776.63–1006.63, quartile 3 (Q3): 1006.63–1330.43, and quartile 4 (Q4): ≥ 1330.43). The baseline characteristics of the four groups according to SII levels were shown in Table 1. Compared with high level of SII group (Q4), patients had a significantly lower follicle count (AFC), E2 on HCG start day, P on HCG start day in other three groups (Q1-Q3).

**Table 1:** Baseline characteristics of participants by quartiles of SII

Characteristics	Quantile 1 (*N* = 242)	Quantile 2 (*N* = 241)	Quantile 3 (*N* = 241)	Quantile 4 (*N* = 242)	*P*-value
Age	28.32 ± 3.18	28.54 ± 3.23	28.20 ± 3.33	28.18 ± 2.92	0.581
Primary infertility	164 (67.8%)	173 (71.8%)	183 (75.9%)	176 (72.7%)	0.254
	78 (32.2%)	68 (28.2%)	58 (24.1%)	66 (27.3%)	
Duration of infertility(y)	3.12 ± 2.00	3.37 ± 1.96	3.21 ± 2.04	3.45 ± 2.19	0.281
Smoking history, n (%)					
No	240 (99.2%)	240 (99.6%)	240 (99.6%)	240 (99.2%)	1.000
Yes	2 (0.8%)	1 (0.4%)	1 (0.4%)	2 (0.8%)	
Gravidity	0.00 (0.00 to 1.00)	0.00 (0.00 to 1.00)	0.00 (0.00 to 1.00)	0.00 (0.00 to 1.00)	0.363
Parity	0.00 (0.00 to 0.00)	0.00 (0.00 to 0.00)	0.00 (0.00 to 0.00)	0.00 (0.00 to 0.00)	0.662
Number of abortions	0.00 (0.00 to 1.00)	0.00 (0.00 to 0.00)	0.00 (0.00 to 0.00)	0.00 (0.00 to 0.00)	0.661
Basal FSH(IU/L)	6.84 ± 1.58	6.74 ± 3.03	6.60 ± 1.39	6.37 ± 1.65	0.060
Basal E2(pg/mL)	55.11 ± 10.20	44.41 ± 20.33	50.26 ± 37.80	46.47 ± 20.89	0.182
Basal LH(IU/L)	7.72 ± 4.01	7.11 ± 4.02	7.71 ± 4.02	7.98 ± 4.69	0.139
Basla T(ng/dl)	0.56 (0.43 to 0.70)	0.55 (0.43 to 0.72)	0.56 (0.42 to 0.70)	0.57 (0.42 to 0.75)	0.997
AMH (ng/ml)	11.16 ± 5.28	11.48 ± 5.48	11.52 ± 5.32	12.27 ± 5.67	0.155
AFC	14.23 ± 4.27	15.07 ± 4.79	16.16 ± 5.28	16.46 ± 5.17	< .001
BMI (Kg/m^2^)					0.680
< 18.5	11 (4.55%)	8 (3.32%)	8 (3.32%)	11 (4.55%)	
18.5–25	144 (59.50%)	128 (53.11%)	143 (59.34%)	132 (54.55%)	
25–30	65 (26.86%)	77 (31.95%)	71 (29.46%)	69 (28.51%)	
≥ 30	22 (9.09%)	28 (11.62%)	19 (7.88%)	30 (12.40%)	
Starting dose of Gn (IU)	188.21 ± 33.11	192.71 ± 39.46	188.68 ± 35.15	188.42 ± 36.61	0.466
Total Gn dose (IU)	1834.29 ± 590.33	1887.87 ± 659.64	1852.45 ± 712.67	1795.18 ± 625.93	0.464
Duration of Gn (day)	9.29 ± 1.72	9.24 ± 1.72	9.28 ± 2.01	9.18 ± 1.48	0.910
Starting dose of GnRH-ant (IU)	0.25 (0.12 to 0.25)	0.25 (0.12 to 0.25)	0.25 (0.12 to 0.25)	0.25 (0.12 to 0.25)	0.258
Total GnRH-ant dose^a^(IU)	1.12 (0.75 to 1.50)	1.00 (0.75 to 1.50)	1.25 (0.88 to 1.50)	1.25 (0.88 to 1.50)	0.155
Duration of GnRH-ant (day)	5.19 ± 2.16	4.82 ± 1.75	5.10 ± 1.87	5.17 ± 1.82	0.121
Triggered with hCG	217(89.67%)	209(86.72%)	206(85.48%)	197(81.40%)	0.072
Triggered with GnRH-a + HCG	25(10.33%)	32(13.28%)	35(14.52%)	45(18.59%)	
E2 on HCG start day (pg/ml)	5769.50 (4257.00 to 8217.00)	5612.00 (4395.00 to 8325.00)	6142.00 (4555.00 to 9117.00)	6871.00 (4805.37 to 9928.00)	0.019
P on HCG start day(ng/ml)	1.25 (0.91 to 1.69)	1.29 (0.94 to 1.70)	1.31 (0.92 to 1.84)	1.42 (1.05 to 2.00)	0.017
LH on HCG start day (IU/L)	2.69 (1.67 to 3.97)	2.65 (1.66 to 3.96)	2.67 (1.75 to 4.77)	2.59 (1.64 to 4.64)	0.907
P on HCG start day(ng/ml)	1.25 (0.91 to 1.69)	1.29 (0.94 to 1.70)	1.31 (0.92 to 1.84)	1.42 (1.05 to 2.00)	0.017
LH on HCG start day (IU/L)	2.69 (1.67 to 3.97)	2.65 (1.66 to 3.96)	2.67 (1.75 to 4.77)	2.59 (1.64 to 4.64)	0.907

Data are expressed as median (interquartile range) for non-normally distributed continuous variables

Data are expressed as mean + SD for normally distributed continuous variables

Categorical variables were expressed in frequency or as a percentage

Abbreviations: *SII* Systemic immune-inflammation index, *BMI* Body mass index, *FSH* Follicle-stimulating hormone, *LH* Luteinizing hormone, *T* Testosterone, *AMH* Anti-müllerian hormone, *P* Progesterone, *AFC* Antral follicle count, *Gn* Gonadotropin, *GnRH-ant* Gonadotropin releasing hormone antagonist, *HCG* Human chorionic gonadotropin, *GnRH-a* Gonadotropin releasing hormone agonist

Comparing the laboratory outcomes, we observed that patients in the lower three SII groups (Q1-Q3) had significantly lower numbers of oocytes retrieved, 2PN cleavage, 2PN and cleavage embryos than those in the highest SII group (Q4). However, the rate of available embryos and blastocyst formation in the three lower SII groups were much higher than that in the highest SII group (Q4) Table 2.

**Table 2:** Comparison of laboratory data between the four groups

Characteristics	Quantile 1 (*N* = 242)	Quantile 2 (*N* = 241)	Quantile 3 (*N* = 241)	Quantile 4 (*N* = 242)	*P*-value
No. of oocytes retrieved(n)	14.30 ± 5.31	14.89 ± 5.63	15.70 ± 6.36	16.80 ± 6.91	< 0.001
No. of 2PN cleavage	10.00 (7.00 to 15.00)	12.00 (8.00 to 15.00)	11.00 (8.00 to 16.00)	13.00 (9.00 to 17.00)	< 0.001
No. of 2PN(n)	10.00 (7.00 to 15.00)	12.00 (8.00 to 15.00)	12.00 (8.00 to 16.00)	13.00 (9.00 to 18.00)	< 0.001
cleavage rate(%)	99.66 (2675/2684)	99.65 (2837/2847)	99.62 (2894/2905)	99.72 (3238/3247)	0.137
No. of cleavage embryos(n)	11.42 ± 5.72	12.18 ± 5.61	12.39 ± 6.24	13.70 ± 6.82	< 0.001
No. of available embryos(n)	7.08 ± 4.22	7.94 ± 4.41	7.84 ± 4.73	7.19 ± 4.19	0.064
Available embryo rate(%)	67 (50 to 82)	70 (55 to 83)	67 (47 to 85)	54 (43 to 67)	< 0.001
High-quality embryos(n)	2.00 (1.00 to 4.00)	3.00 (1.00 to 5.00)	2.00 (1.00 to 5.00)	3.00 (1.00 to 6.00)	0.329
Blastocyst formation rate(%)	56.03(1291/2304)	60.11(1495/2487)	58.78(1475/2868)	57.04(1636/2868)	0.019
OHSS rate(%)	3.75(9/242)	5.8(14/241)	9.1 (22/241)	6.6 (16/242)	0.106

Data are expressed as median (interquartile range) for non-normally distributed continuous variables

Data are expressed as mean + SD for normally distributed continuous variables

Categorical variables were expressed in frequency or as a percentage

*Abbreviations*: *2PN* Two pronucleus, *OHSS* Ovarian hyperstimulation syndrome

### Univariate analysis

The results of univariate analysis were shown in Table 3, Supplementary Tables 1 & 2. Table 3 showed that anti-mullerian hormone (AMH), LH, AFC were positively correlated with numbers of available embryos. We also found that basal FSH, body mass index (BMI) ≥ 30, starting dose of Gn, duration of Gn were negatively associated with numbers of available embryos, whereas age, duration of infertility, basal E2, SII and Log SII were not associated with numbers of available embryos. The univariate analysis of available embryo rate and high-quality embryos were presented in Supplementary Tables 1 & 2.

**Table 3:** The results of uivariate analysis

No. of available embryos	Statistics	β(95%CI), *P* value
Age	28.31 ± 3.17	-0.05 (-0.13, 0.04), 0.298
Duration of infertility(y)	3.29 ± 2.05	-0.10 (-0.24, 0.03), 0.133
BMI(Kg/m^2^)		
< 18.5	38 (3.93%)	0
18.5–25	547 (56.63%)	-0.35 (-1.78, 1.07), 0.626
25–30	282 (29.19%)	-1.39 (-2.86, 0.07), 0.063
≥ 30	99 (10.25%)	-2.71 (-4.33, -1.08), 0.001
AMH(ng/ml)		
Low (< 7.44)	321 (33.23%)	0
Middle (7.44–10.66)	323 (33.44%)	1.43 (0.75, 2.10), < 0.0001
High (≥ 10.66)	322 (33.33%)	1.98 (1.30, 2.66), < 0.0001
Basal FSH(IU/L)	6.64 ± 2.02	-0.20 (-0.34, -0.06), 0.004
Basal E2(pg/mL)	49.07 ± 57.43	0.00 (-0.00, 0.01), 0.861
Basal LH(IU/L)	7.63 ± 4.20	0.12 (0.06, 0.19), 0.0003
AFC	15.48 ± 4.96	0.40 (0.35, 0.45), < 0.0001
Starting dose of Gn(IU)	189.50 ± 36.14	-0.02 (-0.03, -0.01), < 0.0001
Duration of Gn(IU)	9.25 ± 1.74	-0.21 (-0.37, -0.05), 0.011
SII	1100.36 ± 463.46	0.00 (-0.00, 0.00), 0.569
SII quartile		
Q1	242 (25.05%)	0
Q2	241 (24.95%)	0.86 (0.08, 1.64), 0.031
Q3	241 (24.95%)	0.76 (-0.02, 1.54), 0.057
Q4	242 (25.05%)	0.11 (-0.68, 0.89), 0.787
SII quartile continuous	1.50 ± 1.12	0.02 (-0.23, 0.27), 0.860
Log SII	6.92 ± 0.41	0.27 (-0.42, 0.95), 0.445

Data is represented as β(95%CI), *P* value

SII: Quantile 1 Quantile 2 Quantile 3 Quantile 4

*Abbreviations*: *SII* Systemic immune-inflammation index, *BMI* Body mass index, *FSH* Follicle-stimulating hormone, *LH* Luteinizing hormone, *T* Testosterone, *AMH* Anti-müllerian hormone, *P* Progesterone, *AFC* Antral follicle count, *Gn* Gonadotropin, *GnRH-ant* Gonadotropin releasing hormone antagonist; HCG human chorionic gonadotropin

### The relationship between SII quartiles and laboratory outcomes

The association between SII quartiles and laboratory outcomes was shown in Table 4. We used univariate linear regression model to evaluate the associations between SII quartiles and laboratory outcomes. Meanwhile, we showed the non-adjusted and adjusted models. In crude model, Log SII showed no correlation with number of available embryos and high-quality embryos, but a negative correlation was found in Log SII and available embryo rate. In the minimally adjusted model (adjusted age, AMH, BMI, AFC), the highest quartiles of SII showed negative associations with number of available embryos (β = -0.72, 95% CI -1.45 to 0.01; *P* for trend = 0.0195), available embryo rate (β = -0.10, 95% CI -0.15 to -0.06; *P* for trend < 0.0001), when compared with the lowest quartiles of SII. No significant associations were observed between any quartiles of SII and high-quality embryos. Consistent results were also observed in the fully adjusted model when adjusted for age, AMH, BMI, AFC, basal FSH, basal E_2_, basal LH, total Gn dose, duration of Gn, E2 on HCG start day, P on HCG start day.

**Table 4:** Association between quartiles of SII and laboratory data among the whole participants (*N* = 966)

Variable	Crude model (β, 95%CI, *P*)	Minimally adjusted model(β, 95%CI, *P*)	Fully adjusted model(β, 95%CI, *P*)
No. of available embryos			
SII quartile			
Q1	Ref	Ref	Ref
Q2	0.86 (0.08, 1.64), 0.032	0.64 (-0.08, 1.36), 0.082	0.74 (0.02, 1.46), 0.046
Q3	0.76 (-0.02, 1.54), 0.058	0.07 (-0.64, 0.79), 0.838	0.12 (-0.60, 0.84), 0.748
Q4	0.11 (-0.68, 0.89), 0.788	-0.72 (-1.45, 0.01), 0.052	-0.71 (-1.44, 0.02), 0.056
Log SII	0.27 (-0.42, 0.95), 0.445	-0.56 (-1.19, 0.08), 0.089	-0.57 (-1.21, 0.07), 0.079
*P* for trenda	0.8601	0.0195	0.0187
Available embryo rate			
SII quartile			
Q1	Ref	Ref	Ref
Q2	0.03 (-0.01, 0.07) 0.163	0.02 (-0.02, 0.07) 0.247	0.03 (-0.02, 0.07) 0.239
Q3	0.01 (-0.03, 0.06) 0.507	0.01 (-0.03, 0.06) 0.525	0.01 (-0.03, 0.06) 0.541
Q4	-0.10 (-0.14, -0.06) < 0.0001	-0.10 (-0.15, -0.06) < 0.0001	-0.11 (-0.15, -0.06) < 0.0001
SII Log	-0.08 (-0.11, -0.04) < 0.0001	-0.08 (-0.12, -0.04) < 0.0001	-0.08 (-0.12, -0.04) < 0.0001
*P* for trenda	< 0.0001	< 0.0001	< 0.0001
High-quality embryos			
SII quartile			
Q1	Ref	Ref	Ref
Q2	0.46 (-0.15, 1.08) 0.136	0.31 (-0.28, 0.89) 0.303	0.33 (-0.25, 0.92) 0.266
Q3	0.54 (-0.07, 1.15) 0.084	0.04 (-0.55, 0.62) 0.903	0.05 (-0.53, 0.64) 0.856
Q4	0.41 (-0.20, 1.02) 0.185	-0.17 (-0.76, 0.42) 0.569	-0.17 (-0.76, 0.43) 0.583
Log SII	0.48 (-0.05, 1.01) 0.0789	-0.09 (-0.60, 0.42) 0.733	-0.09 (-0.60, 0.43) 0.745
*P* for trenda	0.182	0.411	0.417

Tests for linear trend were conducted by assigning median values of each quartile of systemic immune-inflammation index as a continuous variable in the models

Crude model: did not adjust other covariants

Minimally adjusted model: adjusted age; AMH; BMI; AFC

Fully adjusted model: adjusted age; Basal FSH; Basal E2; Basal LH; AMH;AFC; Total Gn dose; Duration of Gn; E2 on HCG start day; P on HCG start day

*Abbreviations*: *CI* Confidence interval, *Ref* Reference, *SII* Systemic immune-inflammation index

### The analyses of non-linear relationship

**Fig. 2:**
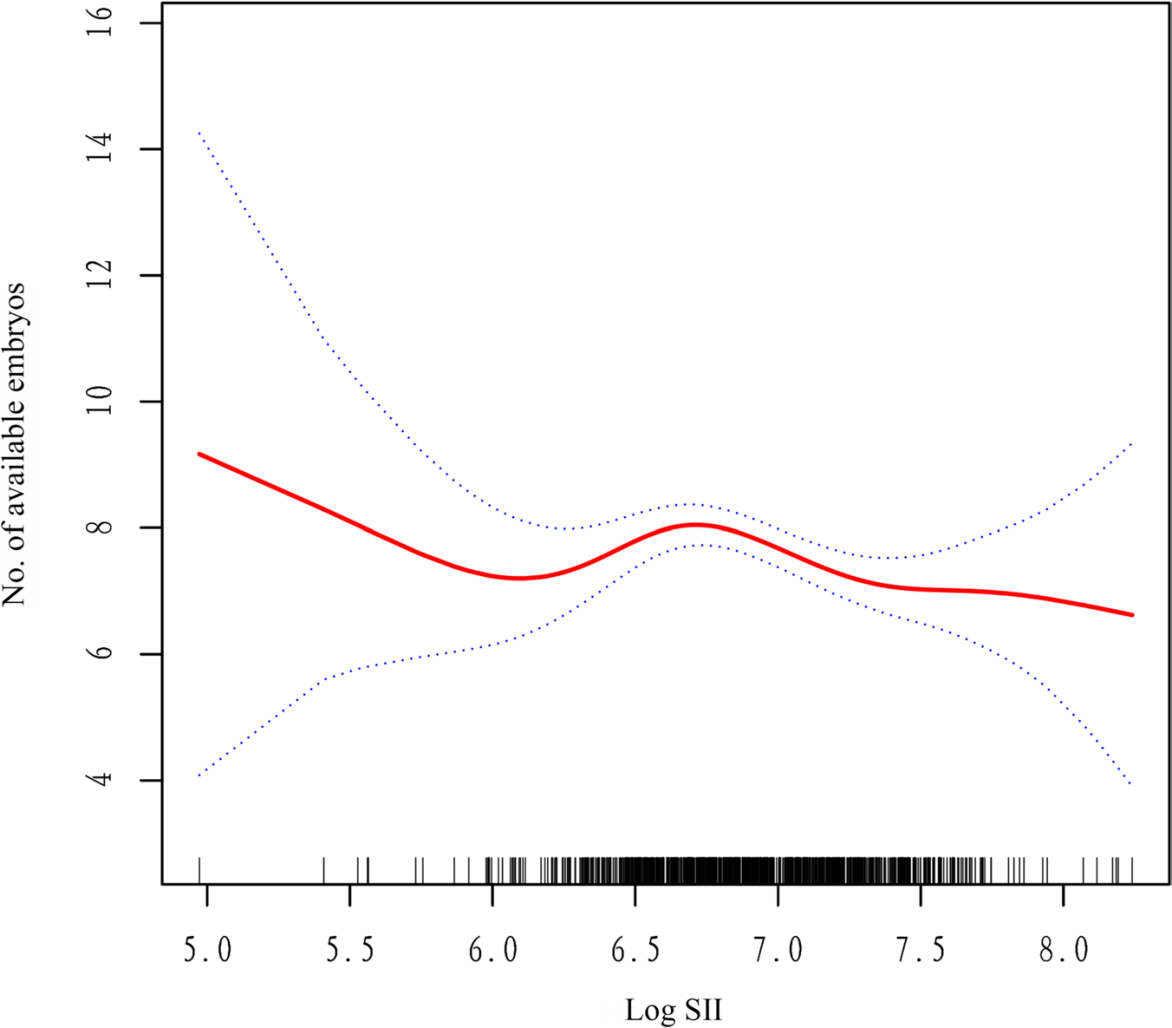
The relationship between number of available embryo and Log SH. A nonlinear relationship between them was detected after adjusting for age, AMH, BMI, AFC, basal FSH, basal E2, basal LH, total Gn dose, duaration of Gn, E2 on HCG start day, P on HCG start day

**Fig. 3:**
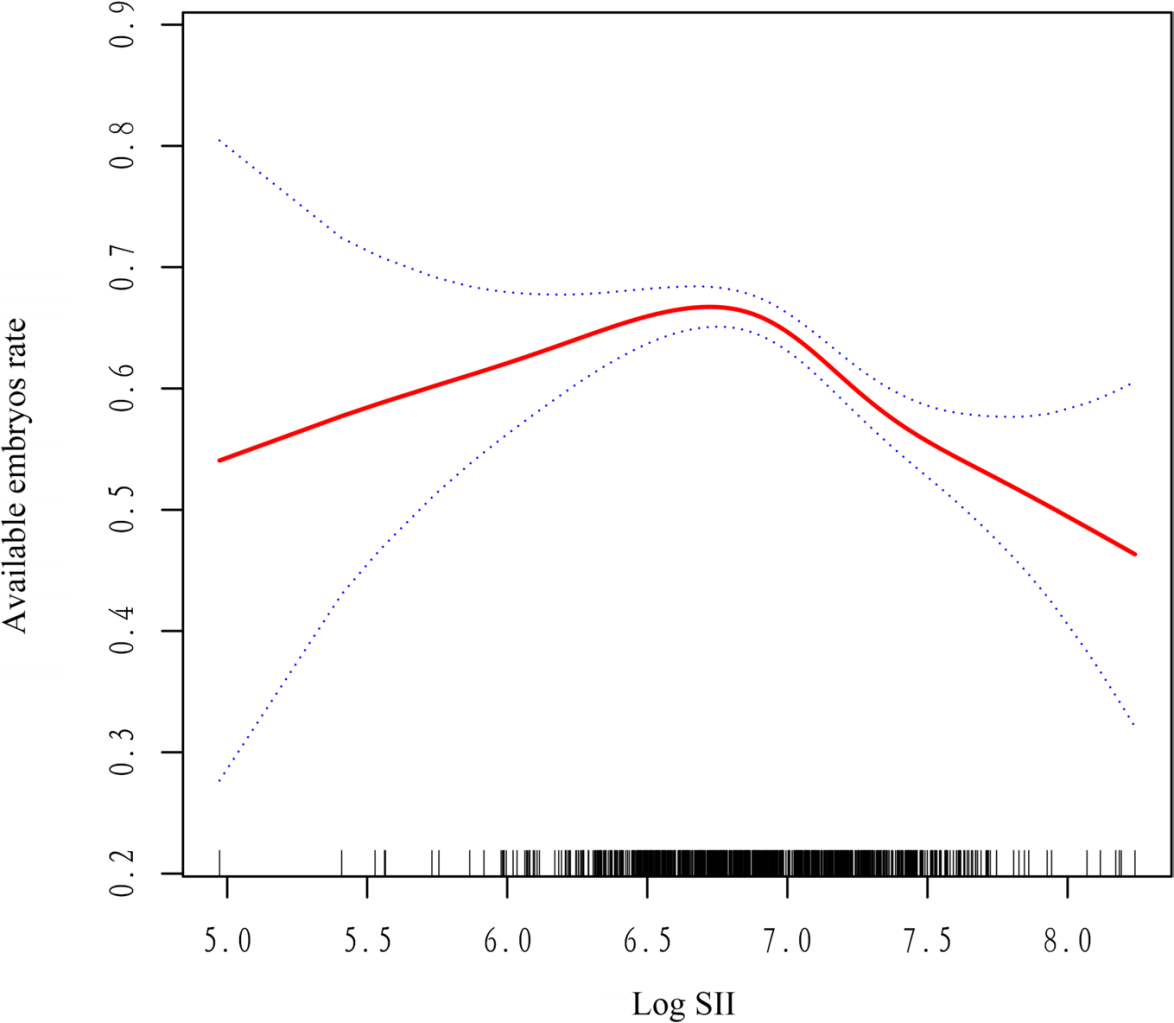
The relationship between available embryos rate and Log SII. A nonlinear relationship between them was detected after adjusting for age, AMH, BMI, AFC, basal FSH, basal E2, basal LH, total Gn dose, duation of Gn, E2 on HCG start day, P on HCG start day

In the present study, we analysed the non-linear relationship between Log SII and outcomes because Log SII was a continuous variable (Fig. 2). We found that the relationship between Log SII and number of available embryos was non-linear (after adjusting age, AMH, BMI, AFC, basal FSH, basal E_2_, basal LH, total Gn dose, duration of Gn, E2 on HCG start day, P on HCG start day). By using a two-piecewise linear regression model, we calculated that the inflection point was 6.72. On the left of the inflection point, the effect size, 95%CI and *P* value were 1.10, − 0.40 to 2.66 and 0.168, respectively (Table 5). However, we also observed a negative relationship between Log SII and number of available embryos on the right side of the inflection point (-1.37, -2.30 to -0.43, 0.004). Consistent results were also observed in the relationship between Log SII and available embryos rate (Supplementary Table 3 & Fig. 3). We also found that while the number of high-quality embryos appeared to decline with an increase in Log SII levels, this observed trend lacked statistical significance (Supplementary Fig. 1). The clinical significance of this finding is that Log SII has a non-linear relationship with the ovarian response and embryo quality in PCOS patients undergoing IVF/ICSI treatment, and that this relationship changes at an inflection point of 6.72.

**Table 5:** The results of two-piecewise linear regression model

Inflection point of Log SII	Effect size (β)	95%CI	*P*
< 6.72	1.10	-0.40 to 2.66	0.168
≥ 6.72	-1.37	-2.30 to -0.43	0.004

*Abbreviations*: *CI* Confidence interval, *SII* Systemic immune-inflammation index

Effect: No. of available embryos, Cause: Log SII

Adjusted: age, sex, BMI, AMH; AFC; BMI; Gn days

### Univariable and multivariate regression analysis of pregnancy outcomes

Table 6 illustrated that both univariate and multivariate logistic regression analyses demonstrate the highest quartiles of SII were negatively associated with biochemical pregnancy rate (BPR), clinical pregnancy rate (CPR), LBR, and IR. Conversely, a positive association was found with EPLR, albeit with no statistically significant difference (*P* > 0.05).

**Table 6:** Univariable and Multivariate regression analysis of Pregnancy outcomes among the whole participants

Variable	Outcome	Univariate analysis		Multivariate analysis	
		OR	*P*	OR	*P*
Biochemical pregnancy rate (%)					
SII quartile					
Q1	58.33(7/12)	Ref		Ref	
Q2	68.75(11/16)	1.57 (0.33, 7.48)	0.570	1.21 (0.23, 6.33)	0.818
Q3	50(3/6)	0.71 (0.10, 5.12)	0.738	0.40 (0.04, 3.88)	0.431
Q4	20(1/5)	0.18 (0.02, 2.12)	0.172	0.05 (0.00, 1.14)	0.061
Clinical pregnancy rate (n%)					
SII quartile					
Q1	58.33(7/12)	Ref		Ref	
Q2	68.75(11/16)	1.57 (0.33, 7.48)	0.570	1.21 (0.23, 6.33)	0.818
Q3	50.00(3/6)	0.71 (0.10, 5.12)	0.738	0.40 (0.04, 3.88)	0.431
Q4	20.00(1/5)	0.18 (0.02, 2.12)	0.172	0.05 (0.00, 1.14)	0.061
Early pregnancy loss rate (n%)					
SII quartile					
Q1	58.33(14/24)	Ref		Ref	
Q2	0(0/11)	-	0.997	-	0.997
Q3	33.33(1/3)	2.20 (0.11, 42.74)	0.602	1.36 (0.05, 36.56)	0.854
Q4	100(1/1)	2.75 (0.14, 55.17)	0.509	2.29 (0.07, 76.10)	0.643
Live birth rate (n%)					
SII quartile					
Q1	50.00(6/12)	Ref		Ref	
Q2	62.50(10/16)	1.67 (0.37, 7.61)	0.509	1.30 (0.26, 6.48)	0.747
Q3	16.67(1/16)	0.20 (0.02, 2.27)	0.194	0.08 (0.00, 1.70)	0.105
Q4	0(0/1)	-	0.992	-	0.994
Implantation rate (n%)					
SII quartile					
Q1	8.33(1/7)	Ref		Ref	
Q2	69.70(23/33)	1.64(0.55,5.01)	0.376	1.34(0.21,8.74)	0.762
Q3	54.55(6/11)	0.86 (0.20, 3.73)	0.834	1.49 (0.21, 19.6)	0.751
Q4	20(2/10)	0.18 (0.02, 0.90)	0.054	0.57 (0.00, 49.5)	0.834

*Abbreviations*: *SII* Systemic immune-inflammation index, *OR*, Odds ratio, *P* value shows significance of entrance in the logistic regression model

SII: Quantile 1 Quantile 2 Quantile 3 Quantile 4

All values are ORs (95% CIs). Values were determined by using logistic regression

## Discussion

SII is a promising inflammatory indicator that integrates data deriving from platelet, neutrophil, and lymphocyte counts, thereby potentially reflects three distinct biological pathways: thrombus formation, the inflammatory response, and the adaptive immune response [28, 29]. In contrast to traditional inflammatory factors, SII better reflects the inflammatory state, and a number of studies have demonstrated that SII reflects the inflammatory state preferably and is more prognostic [20, 30, 31]. In addition, SII is a noninvasive, easy-to-access and low-cost method, which is also universally available. Clinical applications are therefore promising.

We hypothesized that SII could be independently associated with IVF outcomes in women with PCOS. To our knowledge, this is the first study that uses data from a cohort study to investigate whether SII is associated with IVF outcomes. In this study, we found that SII was negatively correlated with the number and rate of available embryos in PCOS patients undergoing IVF/ICSI treatment, after adjusting for confounding factors. This suggests that SII may be a useful marker for predicting the ovarian response and embryo quality in this population. The clinical significance of this finding is that SII could be used as a screening tool for individualizing the treatment protocol and improving the pregnancy rate in PCOS patients.

Prior research has indicated that patients with PCOS exhibit significantly higher concentrations of inflammatory cells in their peripheral blood, accompanying these altered leukocyte counts, the levels of some inflammatory factors, such as serum C-reactive protein (CRP) [32], high sensitivity C-reactive protein (hs-CRP) [33] were also found to be significantly increased in the peripheral blood of PCOS patients. Our study offers the opportunity to simultaneously assess the association between each of the three blood cells (including platelets, neutrophils, and lymphocytes) and IVF outcomes. The SII was divided into quartiles, and comparison across the four groups revealed significant differences in several baseline characteristics and laboratory outcomes. Specifically, patients in the highest SII quartile (Q4) had higher AFC, E_2_, and P levels on the day of HCG start compared to the lower three SII quartiles (Q1-Q3). Furthermore, our analysis demonstrated that women in the lower SII quartiles had a higher rate of available embryos and blastocyst formation compared with those in the highest SII quartile. This suggested of a possible negative correlation between SII and ART outcomes. Similarly, our results showed no significant correlation between the Log SII and the number of available embryos, but a negative correlation was identified between the Log SII and the available embryo rate. These findings were consistent with previous research. While moderate inflammation was a physiological norm during follicle genesis and ovulation, aberrant inflammation may lead to compromised oocyte quality, precipitate oligo-anovulation, and subsequently contribute to infertility [34]. Immunohistochemical staining revealed extensive infiltration of macrophages and lymphocytes throughout the ovaries at PCOS cases [35], which was indicative of a persistent low-grade chronic inflammation.

Inflammation is deemed a key characteristic of endothelial dysfunction and atherosclerosis [36], and is also associated with prominent features of PCOS, including insulin resistance and cardiovascular diseases [37]. Orio et al. has demonstrated the elevations of white cell count in PCOS women [38]. In PCOS patients, the intercellular adhesion molecule (ICAM)-1, tumor necrosis factor (TNF)-α, and monocyte chemoattractant protein (MCP)-1 have been detected in higher concentration [39]. These parameters are indicative of an inflammatory state within the body. PCOS patients have higher mean platelet volume (MPV) and neutrophil to lymphocyte ratio compared with healthy women [40]. Leukocytosis, neutrophilia, and platelet aggregation are frequently observed phenomena in the blood profiles of patients with PCOS [14]. Increased numbers of lymphocytes and macrophages secrete more inflammatory cytokines, such as TNF-α, lymphokine and Interleukin (IL)-6, which can in turn strengthen the secretory function of these two kinds of cells to produce more inflammatory cytokines [41].

As a heterogeneous syndrome characterized by endocrine abnormalities and metabolic dysfunction, PCOS can affect every stage of reproduction, including folliculogenesis and implantation [42]. Previously, PCOS has been associated with adverse effects on ovarian response and IVF outcomes, as well as a higher rate of miscarriages [43]. The results of our multivariate logistic regression analyses indicate that the highest SII quartile is negatively associated with BPR, CPR, LBR, and IR. One hypothesis is that SII serves as an indicator of systemic immune activation, which has previously been linked to compromised implantation and placentation processes. Another hypothesis is that SII could be a marker for endometrial dysfunction, a condition known to influence endometrial receptivity and the interactions between the embryo and endometrium. This suggests that women with PCOS and higher SII may face greater challenges in achieving successful pregnancy and live birth outcomes.

Interestingly, we observed a non-linear relationship between the SII and number of available embryos, with a negative relationship seen to the right of the inflection point. This non-linearity underscores the complexity of the relationship between inflammation and fertility outcomes in PCOS patients. The possible mechanisms behind the non-linear relationship between SII and reproductive outcomes in PCOS patients are not fully understood, but several hypotheses can be proposed. One possible explanation is that SII reflects the balance between immune activation and suppression in the body, which has been shown to play a crucial role in implantation and pregnancy maintenance. Another possible explanation is that SII reflects the threshold effect of inflammation on ovarian function and embryo quality in PCOS patients, which has been shown to vary depending on the degree of inflammation. A third possible explanation is that SII reflects the interaction between inflammation and other factors that affect reproductive outcomes in PCOS patients, such as oxidative stress, insulin resistance, or hormonal imbalance.

Notably, while our study indicated a trend of declining number of high-quality embryos with increasing Log SII levels, this trend lacked statistical significance, highlighting an area for future research to explore.

Our study possessed several merits. Foremost among these was that our research emerged as the inaugural cohort study delineating the association of SII with ART outcomes in PCOS-afflicted women. Furthermore, we adjusted for potential confounders to ensure the reliability of our results. However, certain limitations were present. The cohort did not have comprehensive data at the time of blood sample collection, missing markers of acute inflammatory state, such as CRP levels, and information on the use of certain medications, a small fresh transfer sample does. While SII shows promise as a biomarker to predict pregnancy outcomes in PCOS patients undergoing IVF/ICSI treatment, it necessitates further validation to ascertain its clinical relevance. Consequently, to establish causality, prospective studies with more extensive sample sizes remain imperative.

## Conclusion

Our research reveals that increased level of SII is correlated with suboptimal reproductive outcomes in females diagnosed with PCOS. Further large-scale prospective studies are still needed to validate our findings.

### Supplementary Information

**Additional file 1: Supplementary  Table 1.  **The results of uivariate analysis. ** Supplementary  Table 2.  **The results of uivariate analysis.** Supplementary Table 3. **The results of two-piecewise linear regression model. 

**Additional file 2: Supplementary fig 1. **The relationship between high-quality embryos and Log SII 

## Data Availability

The data underlying this article will be shared on reasonable request to the corresponding author.

## References

[1] Dumesic DA, Oberfield SE, Stener-Victorin E, Marshall JC, Laven JS, Legro RS (2015). Scientific Statement on the Diagnostic Criteria, Epidemiology, Pathophysiology, and Molecular Genetics of Polycystic Ovary Syndrome. Endocr Rev..

[2] March WA, Moore VM, Willson KJ, Phillips DI, Norman RJ, Davies MJ (2010). The prevalence of polycystic ovary syndrome in a community sample assessed under contrasting diagnostic criteria. Hum Reprod..

[3] Rotterdam EA-SPcwg (2004). Revised 2003 consensus on diagnostic criteria and long-term health risks related to polycystic ovary syndrome (PCOS). Hum Reprod.

[4] Wang Y, Yang Q, Wang H, Zhu J, Cong L, Li H (2021). NAD+ deficiency and mitochondrial dysfunction in granulosa cells of women with polycystic ovary syndromedouble dagger. Biol Reprod.

[5] Mizgier M, Jarzabek-Bielecka G, Wendland N, Jodlowska-Siewert E, Nowicki M, Brozek A, et al. Relation between Inflammation, Oxidative Stress, and Macronutrient Intakes in Normal and Excessive Body Weight Adolescent Girls with Clinical Features of Polycystic Ovary Syndrome. Nutrients. 2021;13(3). 10.3390/nu13030896 10.3390/nu13030896 PMC8001803 33801995

[6] Patel S (2018). Polycystic ovary syndrome (PCOS), an inflammatory, systemic, lifestyle endocrinopathy. J Steroid Biochem Mol Biol.

[7] Stener-Victorin E, Deng Q (2021). Epigenetic inheritance of polycystic ovary syndrome - challenges and opportunities for treatment. Nat Rev Endocrinol.

[8] Ma X, Wang Z, Zhang C, Bian Y, Zhang X, Liu X (2022). Association of SNPs in the FK-506 binding protein (FKBP5) gene among Han Chinese women with polycystic ovary syndrome. BMC Med Genomics.

[9] Rajkumar E, Ardra A, Prabhu G, Pandey V, Sundaramoorthy J, Manzoor R (2022). Polycystic ovary syndrome: An exploration of unmarried women's knowledge and attitudes. Heliyon..

[10] Abraham Gnanadass S, Divakar Prabhu Y, Valsala Gopalakrishnan A (2021). Association of metabolic and inflammatory markers with polycystic ovarian syndrome (PCOS): an update. Arch Gynecol Obstet..

[11] Qin L, Xu W, Li X, Meng W, Hu L, Luo Z (2016). Differential Expression Profile of Immunological Cytokines in Local Ovary in Patients with Polycystic Ovarian Syndrome: analysis by Flow Cytometry. Eur J Obstet Gynecol Reprod Biol.

[12] Velez LM, Seldin M, Motta AB (2021). Inflammation and reproductive function in women with polycystic ovary syndromedagger. Biol Reprod..

[13] Yilmaz MA, Duran C, Basaran M (2016). The mean platelet volume and neutrophil to lymphocyte ratio in obese and lean patients with polycystic ovary syndrome. J Endocrinol Invest.

[14] Dasanu CA, Clark BA, Ichim TE, Alexandrescu DT (2011). Polycystic ovary syndrome: focus on platelets and prothrombotic risk. South Med J.

[15] Chen JH, Zhai ET, Yuan YJ, Wu KM, Xu JB, Peng JJ (2017). Systemic immune-inflammation index for predicting prognosis of colorectal cancer. World J Gastroenterol.

[16] Jomrich G, Paireder M, Kristo I, Baierl A, Ilhan-Mutlu A, Preusser M (2021). High Systemic Immune-Inflammation Index is an Adverse Prognostic Factor for Patients With Gastroesophageal Adenocarcinoma. Ann Surg.

[17] Tanacan A, Uyanik E, Unal C, Beksac MS (2020). A cut-off value for systemic immune-inflammation index in the prediction of adverse neonatal outcomes in preterm premature rupture of the membranes. J Obstet Gynaecol Res.

[18] Nie D, Gong H, Mao X, Li Z (2019). Systemic immune-inflammation index predicts prognosis in patients with epithelial ovarian cancer: A retrospective study. Gynecol Oncol.

[19] Huang Y, Chen Y, Zhu Y, Wu Q, Yao C, Xia H (2021). Postoperative Systemic Immune-Inflammation Index (SII): A Superior Prognostic Factor of Endometrial Cancer. Front Surg..

[20] Hu B, Yang XR, Xu Y, Sun YF, Sun C, Guo W (2014). Systemic immune-inflammation index predicts prognosis of patients after curative resection for hepatocellular carcinoma. Clin Cancer Res.

[21] Wang BL, Tian L, Gao XH, Ma XL, Wu J, Zhang CY (2016). Dynamic change of the systemic immune inflammation index predicts the prognosis of patients with hepatocellular carcinoma after curative resection. Clin Chem Lab Med.

[22] Yang R, Chang Q, Meng X, Gao N, Wang W (2018). Prognostic value of Systemic immune-inflammation index in cancer: A meta-analysis. J Cancer.

[23] Hanahan D, Weinberg RA (2011). Hallmarks of cancer: the next generation. Cell.

[24] Zhang J, Sun YF, Xu YM, Shi BJ, Han Y, Luo ZY (2021). Effect of Endometrium Thickness on Clinical Outcomes in Luteal Phase Short-Acting GnRH-a Long Protocol and GnRH-Ant Protocol. Front Endocrinol (Lausanne).

[25] Chen X, Zhang J, Wu X, Cao S, Zhou L, Wang Y (2014). Trophectoderm morphology predicts outcomes of pregnancy in vitrified-warmed single-blastocyst transfer cycle in a Chinese population. J Assist Reprod Genet.

[26] Xu M, Chen R, Liu L, Liu X, Hou J, Liao J (2021). Systemic immune-inflammation index and incident cardiovascular diseases among middle-aged and elderly Chinese adults: The Dongfeng-Tongji cohort study. Atherosclerosis.

[27] Xu X, Yang A, Han Y, Wang W, Hao G, Cui N (2022). The Association Between Serum Estradiol Levels on hCG Trigger Day and Live Birth Rates in Non-PCOS Patients: A Retrospective Cohort Study. Front Endocrinol (Lausanne).

[28] Azab B, Zaher M, Weiserbs KF, Torbey E, Lacossiere K, Gaddam S (2010). Usefulness of neutrophil to lymphocyte ratio in predicting short- and long-term mortality after non-ST-elevation myocardial infarction. Am J Cardiol.

[29] Kurtul A, Ornek E (2019). Platelet to Lymphocyte Ratio in Cardiovascular Diseases: A Systematic Review. Angiology.

[30] Atalay F, Kars A, Topal K, Yavuz Z (2022). Systemic immune inflammation index in patients with recurrent aphthous stomatitis. Braz J Otorhinolaryngol.

[31] Nam KW, Kwon HM, Jeong HY, Park JH, Kwon H (2022). Systemic immune-inflammation index is associated with white matter hyperintensity volume. Sci Rep.

[32] Moin ASM, Sathyapalan T, Diboun I, Elrayess MA, Butler AE, Atkin SL (2021). Metabolic consequences of obesity on the hypercoagulable state of polycystic ovary syndrome. Sci Rep.

[33] Nehir Aytan A, Bastu E, Demiral I, Bulut H, Dogan M, Buyru F (2016). Relationship between hyperandrogenism, obesity, inflammation and polycystic ovary syndrome. Gynecol Endocrinol.

[34] Boots CE, Jungheim ES (2015). Inflammation and Human Ovarian Follicular Dynamics. Semin Reprod Med.

[35] Xiong YL, Liang XY, Yang X, Li Y, Wei LN (2011). Low-grade chronic inflammation in the peripheral blood and ovaries of women with polycystic ovarian syndrome. Eur J Obstet Gynecol Reprod Biol.

[36] Hoffman M, Blum A, Baruch R, Kaplan E, Benjamin M (2004). Leukocytes and coronary heart disease. Atherosclerosis.

[37] Duleba AJ, Dokras A (2012). Is PCOS an inflammatory process?. Fertil Steril.

[38] Orio F, Palomba S, Cascella T, Di Biase S, Manguso F, Tauchmanova L (2005). The increase of leukocytes as a new putative marker of low-grade chronic inflammation and early cardiovascular risk in polycystic ovary syndrome. J Clin Endocrinol Metab.

[39] Hu X, Zhang H, Zhuang L, Jin G, Yang Q, Li M (2020). Ubiquitin-Fold Modifier-1 Participates in the Diabetic Inflammatory Response by Regulating NF-kappaB p65 Nuclear Translocation and the Ubiquitination and Degradation of IkappaBalpha. Drug Des Devel Ther.

[40] Mannaerts D, Faes E, Gielis J, Van Craenenbroeck E, Cos P, Spaanderman M (2018). Oxidative stress and endothelial function in normal pregnancy versus pre-eclampsia, a combined longitudinal and case control study. BMC Pregnancy Childbirth.

[41] Diamanti-Kandarakis E, Paterakis T, Alexandraki K, Piperi C, Aessopos A, Katsikis I (2006). Indices of low-grade chronic inflammation in polycystic ovary syndrome and the beneficial effect of metformin. Hum Reprod.

[42] Inal HA, Yilmaz N, Gorkem U, Oruc AS, Timur H (2016). The impact of follicular fluid adiponectin and ghrelin levels based on BMI on IVF outcomes in PCOS. J Endocrinol Invest.

[43] Rittenberg V, Seshadri S, Sunkara SK, Sobaleva S, Oteng-Ntim E, El-Toukhy T (2011). Effect of body mass index on IVF treatment outcome: an updated systematic review and meta-analysis. Reprod Biomed Online.

