# Supplementary material for: The association between systemic immune-inflammation index and in vitro fertilization outcomes in women with polycystic ovary syndrome: a cohort study
Source: J Ovarian Res. 2023 Dec 13;16:236. doi: 10.1186/s13048-023-01321-z (PMC10717301; doi:10.1186/s13048-023-01321-z)
Supplement: Supplementary file 1 — Additional file 1: Supplementary Table 1. The results of uivariate analysis. Supplementary Table 2. The results of uivariate analysis. Supplementary Table 3. The results of two-piecewise linear regression model. [file 13048_2023_1321_MOESM1_ESM.docx]

Supplementary Table

Supplementary Table1 The results of uivariate analysis

| Available embryo rate | Statistics | β(95%CI), *P* value |
| --- | --- | --- |
| Age | 28.3 ± 3.2 | -0.0 (-0.0, 0.0) 0.900 |
| Duration of infertility(y) | 3.3 ± 2.0 | 0.0 (-0.0, 0.0) 0.586 |
| BMI(Kg/m^2^) |  |  |
| <18.5 | 38 (3.9%) | 0 |
| 18.5-25 | 546 (56.5%) | 0.0 (-0.1, 0.1) 0.567 |
| 25-30 | 283 (29.3%) | 0.0 (-0.0, 0.1) 0.268 |
| ≥30 | 99 (10.2%) | 0.0 (-0.1, 0.1) 0.754 |
| AMH(ng/ml) |  |  |
| Low (<7.44) | 321 (33.23%) | 0 |
| Middle (7.44-10.66) | 323 (33.44%) | -0.0 (-0.1, 0.0) 0.246 |
| High (≥10.66) | 322 (33.33%) | -0.0 (-0.0, 0.0) 0.781 |
| Basal FSH(IU/L) | 6.6 ± 2.0 | 0.0 (-0.0, 0.0) 0.603 |
| Basal E2(pg/mL) | 49.1 ± 57.4 | -0.0 (-0.0, 0.0) 0.427 |
| Basal LH(IU/L) | 7.6 ± 4.2 | 0.0 (-0.0, 0.0) 0.498 |
| AFC | 15.5 ± 5.0 | -0.0 (-0.0, 0.0) 0.558 |
| Starting dose of Gn(IU) | 189.5 ± 36.1 | 0.0 (-0.0, 0.0) 0.789 |
| Duration of Gn(IU) | 5.1 ± 1.9 | 0.0 (-0.0, 0.0) 0.243 |
| SII | 1100.4 ± 463.5 | -0.0 (-0.0, -0.0) <0.001 |
| SII quartile |  |  |
| Q1 | 242 (25.1%) | 0 |
| Q2 | 241 (24.9%) | 0.0 (-0.0, 0.1) 0.162 |
| Q3 | 241 (24.9%) | 0.0 (-0.0, 0.1) 0.507 |
| Q4 | 242 (25.1%) | -0.1 (-0.1, -0.1) <0.001 |
| SII quartile continuous | 1.5 ± 1.1 | -0.0 (-0.0, -0.0) <0.001 |
| Log SII | 6.9 ± 0.4 | -0.1 (-0.1, -0.0) <0.001 |

Supplementary Table2 The results of uivariate analysis

| High-quality embryos | Statistics | β(95%CI), *P* value |
| --- | --- | --- |
| Age | 28.3 ± 3.2 | -0.0 (-0.1, 0.0) 0.451 |
| Duration of infertility(y) | 3.3 ± 2.0 | -0.0 (-0.1, 0.1) 0.730 |
| BMI(Kg/m^2^) |  |  |
| <18.5 | 38 (3.9%) | 0 |
| 18.5-25 | 546 (56.5%) | -0.6 (-1.7, 0.6) 0.320 |
| 25-30 | 283 (29.3%) | -1.2 (-2.4, -0.1) 0.040 |
| ≥30 | 99 (10.2%) | -1.9 (-3.2, -0.7) 0.003 |
| AMH(ng/ml) |  |  |
| Low (<7.44) | 321 (33.23%) | 0 |
| Middle (7.44-10.66) | 323 (33.44%) | 0.7 (0.2, 1.2) 0.012 |
| High (≥10.66) | 322 (33.33%) | 1.1 (0.6, 1.7) <0.001 |
| Basal FSH(IU/L) | 6.6 ± 2.0 | -0.1 (-0.2, -0.0) 0.045 |
| Basal E2(pg/mL) | 49.1 ± 57.4 | 0.0 (-0.0, 0.0) 0.946 |
| Basal LH(IU/L) | 7.6 ± 4.2 | 0.1 (0.0, 0.1) 0.007 |
| AFC | 15.5 ± 5.0 | 0.3 (0.2, 0.3) <0.001 |
| Starting dose of Gn(IU) | 189.5 ± 36.1 | -0.0 (-0.0, -0.0) <0.001 |
| Duration of Gn(IU) | 9.2 ± 1.7 | -0.2 (-0.3, -0.0) 0.016 |
| SII | 1100.4 ± 463.5 | 0.0 (-0.0, 0.0) 0.081 |
| SII quartile |  |  |
| Q1 | 242 (25.1%) | 0 |
| Q2 | 241 (24.9%) | 0.5 (-0.1, 1.1) 0.136 |
| Q3 | 241 (24.9%) | 0.5 (-0.1, 1.2) 0.084 |
| Q4 | 242 (25.1%) | 0.4 (-0.2, 1.0) 0.185 |
| SII quartile continuous | 1.5 ± 1.1 | 0.1 (-0.1, 0.3) 0.182 |
| Log SII | 6.9 ± 0.4 | 0.5 (-0.1, 1.0) 0.079 |

Supplementary Table3 The results of two-piecewise linear regression model

| Inflection point of SII | Effect size (β) | 95%CI | *P* |
| --- | --- | --- | --- |
| <6.83 | 0.07 | -0.01 to 0.15 | 0.0598 |
| ≥6.83 | -0.19 | -0.25 to -0.12 | 0.0001 |
