# Supplementary figures and images for: The association between systemic immune-inflammation index and in vitro fertilization outcomes in women with polycystic ovary syndrome: a cohort study
Source: J Ovarian Res. 2023 Dec 13;16:236. doi: 10.1186/s13048-023-01321-z (PMC10717301; doi:10.1186/s13048-023-01321-z)

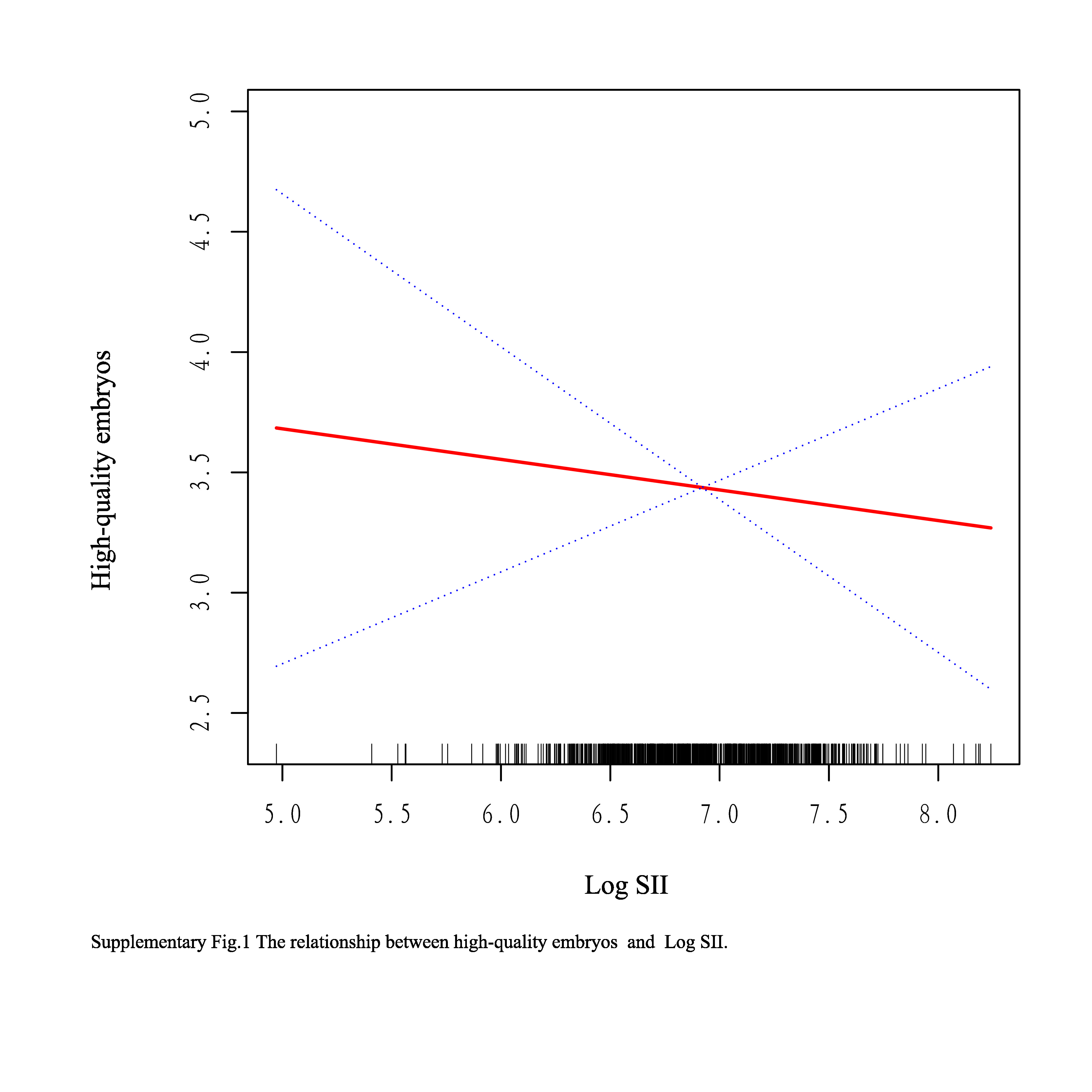

Supplement: Supplementary file 2 — Additional file 2: Supplementary fig 1. The relationship between high-quality embryos and Log SII [file 13048_2023_1321_MOESM2_ESM.tif]
